# Proliferative Verrucous Leukoplakia of the Gingiva: An Early Lesion Refractory to Surgical Excision

**DOI:** 10.1155/2019/5785060

**Published:** 2019-10-22

**Authors:** Dhanushka Leuke Bandara, Primali Rukmal Jayasooriya, Ruwan Duminda Jayasinghe

**Affiliations:** ^1^Department of Oral Medicine & Periodontology, Faculty of Dental Sciences, University of Peradeniya, Peradeniya 20400, Sri Lanka; ^2^Department of Oral Pathology, Faculty of Dental Sciences, University of Peradeniya, Peradeniya 20400, Sri Lanka

## Abstract

This report describes a case of proliferative verrucous leukoplakia (PVL) of the gingiva with no discernible aetiology, which presented in a 36-year-old female. The initial nonscrapable gingival lesion was treated with CO_2_ laser ablation, and the histopathological evaluation was carried out. The presence of koilocytic cells in the superficial epithelium led to immunohistochemical investigations with p16 antibody, which showed strong nuclear positivity and slight cytoplasmic positivity in >50% of the cells with >25% confluency. However, it was not possible to confirm the presence of HPV infection with further investigations due to logistic reasons. The lesion recurred twice within a short time despite the surgical resection following the first recurrence. Thus, this paper presents a case of proliferative verrucous leukoplakia, which demonstrated a significant resistance to routine treatment protocols recommended in the management of such lesions.

## 1. Introduction

White lesions in the oral cavity could represent a variety of aetiological factors including increased or abnormal keratin production in the oral epithelium, which is denoted by the term “keratosis” [1].

In addition to keratotic lesions that occur due to tobacco use, they may also occur due to frictional, chemical, or thermal irritations as well as due to inflammation. Frictional keratosis is usually seen in areas of recurring mild mechanical trauma or irritation while chemical keratosis may occur as the result of the compounds in smokeless tobacco, certain toothpastes, inappropriately used acidic medications, and alkaline liquids [2, 3]. Usually, keratotic lesions cannot be scrapped off and may differ in texture depending on the cause [3].

Oral leukoplakia (OL) is defined as a “white plaque of questionable risk having excluded (other) known diseases or disorders that carry no increased risk for cancer” [4]. Proliferative verrucous leukoplakia (PVL) is a rare form of oral leukoplakia with a malignant transformation rate of 70% [5]. It develops initially as a white plaque of hyperkeratosis that eventually becomes a multifocal disease with confluent, exophytic, and proliferative features showing different degrees of dysplasia. PVL is more commonly a disease of the elderly females which can develop in both tobacco users and nontobacco users [5, 6].

Recently, Woo et al. have reported a subset of lesions, with the majority clinically presenting as OL, harbouring high-risk HPV subtypes and positive for p16 [7]. In addition, clinicopathological spectrums of these lesions are similar to tobacco-induced leukoplakia that occurs more often in adult males on the tongue and floor of the mouth [7]. According to Barasch et al., when interpreting p16 positivity, the presence of p16 positivity in >50% of cells with >25% confluence or >70% positivity could be considered as the threshold to determine a positive reaction [8].

This case report describes a rare case of early stage of PVL with no apparent aetiology that developed in a relatively young patient.

## 2. Case Presentation

A 36-year-old female presented with an asymptomatic whitish lesion, on the gingiva in relation to 13, 14, and 15. The lesion had existed for more than one year in duration which had initiated during her second pregnancy. She was otherwise healthy except for having mild hypochromic microcytic anaemia due to being a carrier for thalassemia trait. However, her serum ferritin levels were within the normal range. Her plaque control measures revealed brushing twice daily with a fluoridated toothpaste although supplementary tools were not used. Furthermore, the patient did not practice any risk habits such as betel chewing, smoking, or alcohol consumption.

**Figure 1:**
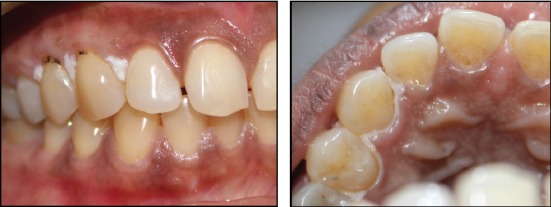
Linear, plaque-like whitish lesion on the palatal and buccal gingiva of 13, 14, and 15.

Intraoral examination revealed a linear, plaque-like whitish lesion on the palatal and buccal free gingiva of 13, 14, and 15 (Figure 1). No verrucous/papillary appearance was evident, and the lesion was nonscrapable.

**Figure 2:**
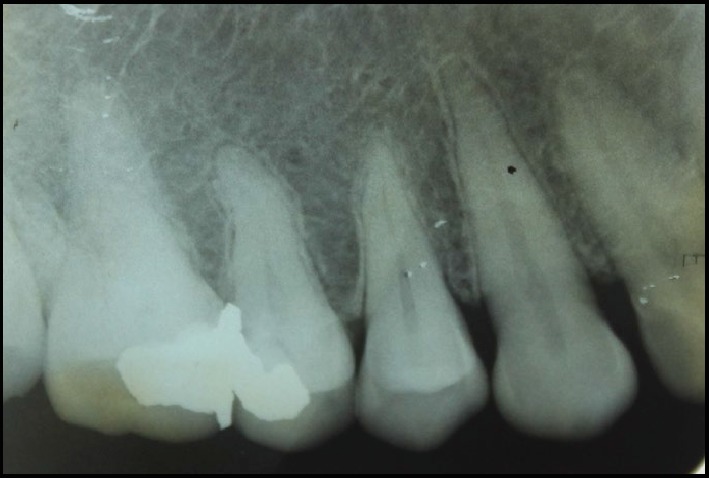
IOPA radiograph of 13–15 region.

She had no other significant extraoral or intraoral findings, and the radiological examination in relation to the site was normal (Figure 2). As baseline investigations, an incisional biopsy was performed together with the necessary haematological investigations.

### 2.1. The Histological Findings

**Figure 3:**
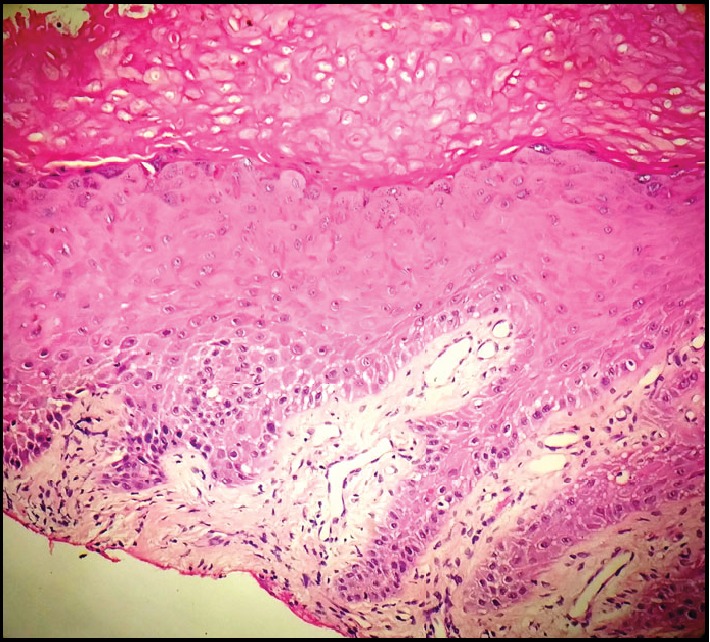
Keratosis with mild epithelial dysplasia under H&E staining.

**Figure 4:**
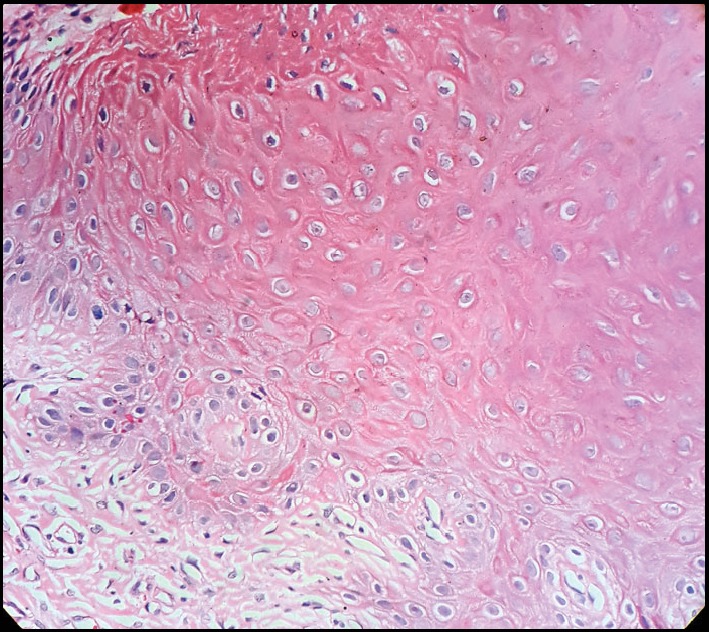
Koilocytic cells under ×40 magnification.

The histopathological analysis revealed a hyperorthokeratinized stratified squamous epithelium which showed cytological atypia in the lower part amounting to mild epithelial dysplasia with numerous koilocytes in the stratum spinosum (Figures 3 and 4).

**Figure 5:**
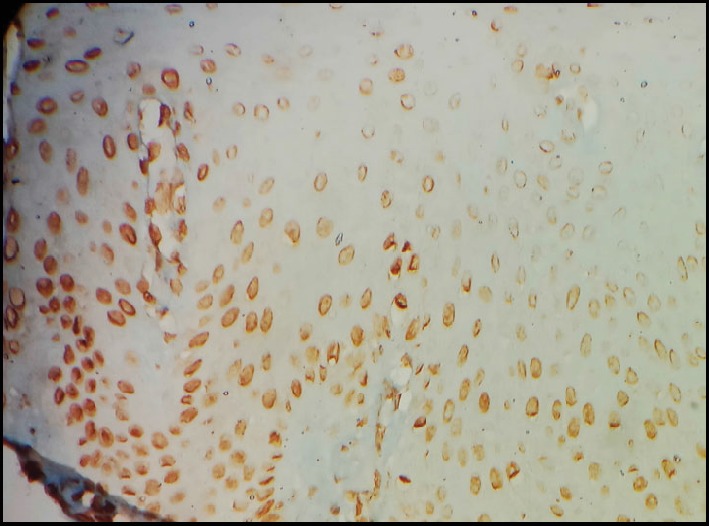
Immunohistochemical assay with p16 showing positive staining in >50% of the cells with >25% cell confluency.

Furthermore, due to the presence of koilocytosis, immunohistochemical evaluation was carried out with p16 antibody which clearly revealed positivity in >50% of the cells with >25% confluency (Figure 5). It is also worthwhile to mention that in the present lesion, nuclear staining was stronger compared to cytoplasmic staining.

However, as p16 is only a surrogate marker, it was not possible to confirm the high-risk HPV infection as the etiological factor for the present lesion due to the absence of adequate amount of fresh tissue. Therefore, considering the histological findings together with the clinical features, early stage of PVL was derived as the possible diagnosis.

### 2.2. Management

Thus, following informing the patient regarding the findings, she was referred for HIV and gynecological screening. The HIV test was negative, and she had no abnormalities detected with the gynecology screening.

However, despite the thorough probing into the possible causative factors including behavioural risk factors and habits, no aetiological factor for this oral lesion was revealed, except for p16 positivity, which could not be confirmed as due to a high-risk HPV infection with further investigations.

For the management of the lesion, resection of the lesion with adequate margins using CO_2_ laser was carried out following improving the oral hygiene. Nevertheless, the lesion reappeared within few weeks at the same sites which led to perform a surgical resection with an adequate margin in the second attempt.

However, the lesion recurred similar to the initial presentation showing its resistance to excision. Thus, within a period of nearly six months, it recurred thrice despite electrosurgical interventions. Considering the diagnosis of PVL, currently, the patient is being followed up with short recall intervals and the lesion remains same with no significant change in size compared to the initial presentation.

## 3. Discussion

As the lesion described in the present report is a rare occurrence, difficulties were encountered when naming the lesion, especially with references to the terms “keratosis” and “leukoplakia.” According to literature, similar lesions have been named as linear gingival keratosis [3] as well as linear gingival leukoplakia [9]. When linear gingival keratosis/leukoplakia reported in literature [10] was compared with the present lesion, it did not show bone erosion. In contrast to lesions reported in literature [9, 10], the present lesion showed dysplasia and p16 positivity. However, in this case, the lesion was diagnosed as an early stage of PVL rather than linear gingival keratosis, due to the fact that it occurred in a female in the gingiva and also as it was refractory to surgical management.

In frictional keratosis histology of the spinous layer often demonstrate intraepithelial edema and occasional vacuolated cells with pyknotic nuclei resembling koilocytes [3]. The diagnosis of frictional keratosis typically consists of a detailed clinical examination, considering the possible oral habits and the agents that may involve in the chronic trauma of the oral mucosa [10]. However, in the present case, no aetiological factor was found indicating frictional irritation. Furthermore, the patient did not reveal any possibility of chemical irritation. A minority of keratotic lesions show dysplastic changes [1]. In this case, although the dysplastic changes were mainly observed in the lower part of the epithelium, dyskeratosis and koilocytosis were mainly observed in the upper layers of the epithelium. Though, it was not possible to confirm the presence of high-risk HPV infection in the present lesion, it would be worthwhile to explore the contribution of HPV in future studies.

When considering the management, PVL is commonly treated with conventional surgery, electrocautery, laser ablation, and cryosurgery [11]. Except in conventional surgery where resection of the lesion is targeted, in other methods, the tissue destruction is obtained via intercellular and extracellular freezing, denaturing lipid–protein complexes, and cell dehydration. There are several advantages of using CO_2_ lasers for oral lesions, particularly less intraoperative bleeding, minimal damage to adjacent tissue, delayed acute inflammatory reaction, and reduced myofibroblast activity, leading to reduced wound contraction and scarring [11]. In addition, though generally lesions that are diagnosed as mild epithelial dysplasia are followed up [1, 4, 12], surgical excision was planned due to aesthetic concerns of the patient.

PVL is a progressive disease and may not respond to traditional treatment [5] similar to the present case which showed a high recurrence rate despite the early interventions carried out in the management.

When there is no obvious aetiology for oral PVL, the lesions may be considered as leukoplakia and managed accordingly [12]. Furthermore, in the present case, histological diagnosis of koilocytic dysplasia was not excluded completely, though the lesion displayed histologic features of HPV infection such as koilocytosis and multinucleated keratinocytes.

## 4. Conclusion

The key feature in the case presented was the high recurrence rate showed despite the various treatment methods. Therefore, the patient should be followed up with close monitoring to detect any further increase in dysplastic changes which could follow with the presence of koilocytic cells and with PVL lesions. In addition, occurrence of early PVL lesions as gingival keratosis should be further explored.
